# Awareness, treatment and control of hypertension among the elderly living in their home in Tunisia

**DOI:** 10.1186/1471-2261-11-65

**Published:** 2011-11-01

**Authors:** Sonia Hammami, Sounira Mehri, Said Hajem, Nadia Koubaa, Mohamed A Frih, Samy Kammoun, Mohamed Hammami, Fathi Betbout

**Affiliations:** 1 Laboratory of Nutrition and Vascular Health, Faculty of Medicine, University of Monastir, Tunisia; 2 Internal Medicine Department, University Hospital "F. Bourguiba" Monastir, Tunisia; 3 National Institute of Public Health Tunis, Tunisia; 4 Nephrology Department, University Hospital "F. Bourguiba" Monastir, Tunisia; 5 Pneumology Department, Geriatric unit, University Hospital H Chaker Sfax, Tunisia; 6 Cardiology Department, University Hospital "F. Bourguiba" Monastir, Tunisia

**Keywords:** Hypertension, Elderly, Prevalence, Awareness, Tunisia, Home living

## Abstract

**Background:**

Hypertension is a cardiovascular disorder rapidly emerging as a major public health problem in developing countries. However, the acknowledgement of the prevalence and the significant impact of hypertension in elderly are very important for health policy. The objective of the present investigation was to evaluate the prevalence, awareness and treatment of hypertension among the elderly living in their home in Tunisia at Monastir City. We also examined the impact of socio-demographic characteristics and known risk factors for high blood pressure.

**Methods:**

A community based sample of 598 non-institutionalized elderly (age ≥ 65 years), was selected using probabilistic multistage cluster sampling.

**Results:**

There was a predominance of female (66%) and mean age was 72.3 ± 7.4 years. The prevalence of hypertension was 52% (n = 311), awareness (81%, n = 252), treatment (78.4%, n = 244) and only 30.7% (n = 75) are correctly treated. The prevalence of hypertension was higher for the female population (55.5%) when compared to males (45%). No urban/rural differences were observed and no difference was observed by educational level. Multiple logistic regression analyses identified a higher body mass index, diabetes mellitus and disability as important correlates of the prevalence of hypertension.

**Conclusion:**

These findings provide important information on the prevalence, awareness and control of hypertension in Monastir City and confirm their association with other cardio-vascular risk factors. Effective public health measures and strategies are needed to improve prevention, diagnosis and access to treatment of this elderly population.

## Background

Hypertension is the leading cause of cardiovascular disease worldwide. Recent studies demonstrate that there is a linear correlation between blood pressure and cardiovascular events [1]. Hypertension is also a major cause of disability, causing an estimated 13% of all deaths in the world [2]. More than 20% of adults are hypertensive with a very poor rate of control and only one third of hypertensive patients treated achieve the correct goal of blood pressure [3]. Age represents a determining factor in the prevalence of arterial hypertension. Epidemiological research has demonstrated that age plays an important role in increasing pressure value, especially systolic blood pressure that tends to increase with age [4,5]. Acknowledgement of the prevalence and the significant impact of hypertension in elderly, on the cardiovascular risk and quality of life are very important for health policy and public health strategy in developing countries.

In Tunisia, the population aged 65 years has exceeded 7% since 2009 [6]. The prevalence of hypertension has not been well studied. The present epidemiological study was performed to estimate the prevalence, awareness and treatment of hypertension in the elderly people living in their home (community dwelling elderly) in Monastir city and to discuss the potential risk factors for hypertension and its association with other cardiovascular risk factors.

## Methods

Data for this study are from a population based survey undertaken in 2008-2009 to investigate social, health behaviours and health status of people aged 65 years and over living in their home in Monastir (center-ouest in Tunisia). Monastir is a governorate of about 494900 inhabitants. The city was divided into 77 sectors and 13 delegations based on the list of the last population census in 2004 (National Institute of Statistics, unpublished data). Ethical approval was granted by the Clinical Research Ethics Committee of the University Hospital of Monastir and Ministry of health. This study was supported by grants from World Health Organisation and the United Nations Population Fund.

### Study Population

We conducted cross-sectional community-based study carried out thought home questionnaires, with a random sampling process of Monastir's non institutionalized elderly individuals (aged more than 65 years). We used a probabilistic multistage cluster sampling technique in two phases. The first consisted in the identification of the districts; eighteen sectors were selected, than we selected randomly a cluster of household with a probability of selection proportional to the size of their population. We identified elderly subjects within the household clusters. At the selected homes one or two resident was interviewed. The participants were previously informed about this study by social assistant of the region to maximize response rates. Three doctors performed a door-to-door survey of the participant households. Any elderly subject refusing to take part in the study will be granted the freedom to do so. The investigator also respects the decision of the old person's attendants and relatives if they express the wish not to allow the elderly person to take part in this study. In all cases, informed consent was obtained from subjects or cohabiting. No response subjects (n = 22) were replaced by others eligible subjects, a next neighbor was chosen in the same way. The parameters used to calculate the sample size were the proportion of elderly, maximum allowed 95% confidence interval with a margin of error no more than 3%. An extra of 50% were added to the sample obtained (n = 400) to cover losses, resulting in n = 600. A total of 598 individuals were investigated. The exclusion criteria were individuals aged less than 65 years. Any elderly present, the day of the visit by investigators, in the surveyed household found to be a visitor or do not usually leave there will be excluded.

### Subject Evaluation

Information was gathered by home-based personal interview using a structured questionnaire with 90 parameters conducted in three parts: standard questions on demographic, socioeconomic information and health behaviors, followed by a physical examination: anthropometric and blood pressure measurements. The questionnaire was translated from French into Arab by the principal investigator and another Tunisian physician and compared before being distributed. Before participation respondents were informed that participation was voluntary, and that they could quit out at any time.

Blood pressure (BP) was measured twice using standard sphygmomanometer, with participants seated after 5 minutes. For analysis we used the mean of the 2 measurements. High BP was defined as systolic blood pressure (SBP) ≥ 140 mmHg and/or diastolic pressure (DBP) ≥ 90 mm Hg [7]. Arterial hypertension (AH) was defined as having high BP or using antihypertensive drug therapy in the previous 2 weeks. Awareness of hypertension reflected a previous diagnosis of hypertension. Controlled hypertension was defined as treated hypertension with SBP < 140 mmHg and DBP < 90 mmHg. Anthropometric measurements were obtained with the participants wearing light clothing and no footwear. Height was measured without shoes, to the nearest 0.1 cm, with subjects standing fully erect on a flat surface, tocks and shoulders flat to the wall and looking straight ahead. Weight was to the nearest 0.1 kg using digital scale. Body Mass Index (BMI) was calculated as weight (in kilograms) divided by squared height (in meters squared) and further. The BMI classified individuals, using World Health Organization criteria, as normal (< 25 kg/m^2^), Overweight (25-29.9 kg/m^2^) and obese (≥ 30 kg/m^2^) [8]. Waist circumference was measured to the nearest centimeter at the level of the midpoint between the inferior border of the ribs and the iliac crest in the midaxillary line, at the end of expiration, using a constant tension tape, directly over light clothing. The waist circumference (WC) normal range was that recommended by the National Cholesterol Education Program ATP III. The WC < 88 cm for women and < 102 cm for men was classified as normal [9]. The diagnosis of diabetes mellitus was established by history or use of glucose-lowering drugs in the previous 2 weeks [10]. Marital status was categorized as married or single, divorced, widows, widowers and never married persons were included in the single group. Smoking status (current smokers or not), and physical activity (walking for at least 1 h per day or not). Education was recorded on four levels scale: (illiterate, kuttabs 'koranic schools', primary, secondary or more). The diagnosis of disability was made by assessing limitations on basic activities of daily living according to Katz's test, was defined as the inability to do one or more of the following without help: bathe, eat, dress, transfer from a bed to a chair, use the toilet, or walk across a small room. Depression symptoms were assessed using a mini-Geriatric Depression Scale mini-GDS, a mini-GDS Scale ≥ 1 was considered indicative of depression [11].

We built in several quality and control measures into our survey protocol, to ensure completeness and comparability of blood pressure and anthropometric measurements and interviewee responses. For this all three doctors, underwent a common training program at the National Institute of Public Health at which a team of cardiologists instructed the investigators regarding measured of BP, weight and height. The questionnaire items were tested for clarity and validity.

### Statistical analysis

The data were collected in a database and transferred to our coordinating center for statistical analysis. Every month a meeting was organised for all participating investigators to discuss the progress of the study. A total of 598 subjects were analysed using SPSS13 statistical software packages. Results are expressed as mean with the corresponding standard deviations. For statistical significance of the difference between two mean values, a Student t test was used. Comparison of frequencies was performed by chi2 tests with the Yates correction when needed. Multivariate analysis between statistically significant variables and hypertension (dichotomous dependent variable) was performed using a logistic regression model. The independent variables were modelled categorically, using dummy terms.

## Results

### Description of study subjects

Table 1 gives a breakdown of the sample characteristics, there is more female than male (66% *VS*. 34%) and mean age was 72.3 ± 7.4 years. Most subjects were aged < 70 years (38%) but only 18% were over 80 years of age. The population under study was predominantly urban (more than three quarters). Only about 14% are from rural sites. Approximately three quarters of the subjects had no formal education and only 5% had secondary or higher education. Most of our population are still married (60%) and 61% are inactive. Only 35% are current smokers. Smoking prevalence is notably high for male (98%) compared to female (2.3%). Health insurance covered more than half of our population (57%).

**Table 1:** Characteristics of elderly population in Monastir City

Patient characteristic	Characteristics of elderly population	N	%
**Sex**	- Men	202	34
	- Women	396	66
**Age (years)**	<70	227	38
	70-74	142	24
	75-79	122	20
	≥ 80	107	18
**Place of residence**	- Urban	516	86
	- Rural	82	14
**Marital Status**	- Married(e)	359	60
	- Single	239	40
**Smoking Status**	-Current smokers	208	35
	-Non smokers	390	65
**Educationnel levels**	- Illiterate	460	77
	- (kuttabs: koranic schools)	72	12
	- Primary	34	6
	- Secondary or higher	32	5
	- Farmer	82	14
**Socio-professional activities**	- Artisans, business	16	3
	- Liberal profession	8	1
	- Workers	127	21
	- Inactifs	365	61

### Hypertension prevalence

The prevalence of hypertension was 52% (n = 311) and was predominant in women (women 55.5%, men 45%, p < 0.01). The prevalence of hypertension increased with age until 80 years then decrease. At age less than 70 years, there are 51.5% compared with 55% of those aged 75 to 79 years and only 45.8% of those aged more than 80 years (Table 2). The mean systolic blood pressure values were 135.2 ± 17.0 mmHg (85 - 21.5 mmHg), while mean diastolic blood pressure values were 80.0 ± 10.2 mmHg (5.5 - 12.5 mmHg). Isolated systolic hypertension was the most common form of hypertension (50.9%) of all hypertensive subjects who had elevated blood pressure at examination, followed by combined systolic and diastolic hypertension (45.7%) and isolated diastolic hypertension (3.4%).

**Table 2:** Prevalence of arterial hypertension according to socio-demographic variable, anthropometric measurements and cardiovascular risk factors of elderly population in Monastir city

Variable	N	Prevalence of Hypertension	**p value **^**b**^
**Gender**			
Female	396	220 (55.5) ^a^	
Male	202	91 (45)	P < 0.05
**Age (years)**			
< 70	227	117 (51.5)	
70-74	142	78 (55)	
75-79	122	67 (55)	
≥ 80	107	49 (45.8)	P < 0.01
**Schooling**			
Illiterate	460	248 (54)	
Kuttab (koranic schools)	72	37 (51.3)	
Primary	34	16 (47)	
Secondary or more	32	10 (31.2)	p = 0.08
**Dependency**			
Intense	57	39 (68.4)	
Moderate	150	86 (57.3)	
Autonomy +	391	186 (47.5)	P < 0.01
**Place of residence**			
Urban	516	270 (52.3)	NS ^c^
Rural	82	41 (50)	
**Depression**			
Mini-GDS < 1	462	232 (50.2)	
Mini-GDS ≥ 1	136	79 (58)	NS
**Marital status**			
Married	359	187 (52)	
Single	239	124 (51.8)	NS
**Physical activity**			
Yes	23	8 (34.7)	
No	575	303 (52.6)	NS
**Diabetes**			
Absent	434	195 (45)	
Present	164	116 (70.7)	P < 0.001
**Overweight (kg/m^2^) (n = 531)**			
BMI< 25	106	31 (29.2)	
BMI: 25-29.9	165	81 (49)	
BMI ≥ 30	260	167 (64.2)	p = 0.000
**Abdominal circumference**			
**(n = 531)**			
Normal	142	47 (33)	
Increased	389	235 (60.4)	P = 0.000

^a ^Figures in parentheses are percentages ^b ^chi2 test ^c ^not statistically significant

The proportion of subjects aware of their elevated blood pressure status was 81% (n = 252), the majority (78.4%, n = 244) of these were taking pharmacological treatment for hypertension. However, only about 30.7% (n = 75) of the treated subjects had their blood pressure controlled to normal levels according to current JNC-VII recommendation [7].

### Socio-demographic and clinical correlates of hypertension

Tables 2 examine the frequency of socio-demographic and cardiovascular risk in normotensive and hypertensive participants. Schooling showed an inverse association but not significantly (p = 0.08) with hypertension, with a prevalence of 54% among analphabetic and 31.2% among those with than secondary education level. No urban/rural differences were observed. Depression, marital status, and self reported physical activity were not correlated with the prevalence of hypertension. Smoking status is negatively but not significantly associated with AH (results not shown).

Those with hypertension had higher BMI, waist circumference, and diabetes (p < 0.05). Regarding disability, the individuals who presented the highest prevalence of hypertension were more dependent. In stepwise multivariable regression analysis, the presence of obesity, diabetes, as well as disability was independently associated with hypertension (Table 3).

**Table 3:** Correlates of prevalent hypertension among the study subjects (results of multiple logistic regression analysis)

Variable	β-Coefficient	Odds ratio	p value
Diabetes	0.81 (0.22) ^c^	2.06; *1.45 - 3.5 *^d^	P < 0.001
BMI ^b^	0.65 (0.20)	2.01; *1.48 - 2.47*	P < 0.01
Dependency	0.63 (0.15)	1.6; *0.9 - 2.7*	P < 0.001

^a ^Age, sex, marital status, region, educational level, physical activity, depression were not statistically significant. ^b ^BMI Body Mass Index. ^c ^Figures in parentheses are standard errors.

^d ^Figures in italics are 95% confidence intervals.

## Discussion

### Prevalence of hypertension

This study provides important new evidence on the prevalence of hypertension in a representative sample of an elderly population in a geographically well defined Mediterranean area of the Tunisia center. Moreover, it has already established socio-demographic variables and drug consumption. A peculiar aspect of our population is the low level of education (89% for illiterates plus subjects with kuttabs education).

We observed that more than 50% of elderly in Monastir city suffer from hypertension. Since the use a BP value of 140/90 mmHg as cut-off, the prevalence of hypertension has increased. These percentage values have also been found in studies carried out in North African countries such as Morocco [13], and other Arab countries such as Egypt [14]. Laouani reported a prevalence rate of 69.3% [15] but in 2000, Kammoun et al. reported in a representative sample of the Tunisian elderly population the prevalence of 32% [16]. The increasing prevalence of hypertension among the elderly population of Monastir city may be attributed to change in lifestyle in the past decade. However, the prevalence of hypertension is still considerably lower than in other industrialised countries such as France (79.8%), England (62%), Greece (65.4%), Spain (62%), Italy (76.3%) and United States (84%) [17-22].

This study showed clear gender differences in the prevalence of hypertension, with women being more likely to be hypertensive than men. The gender differences in the prevalence of hypertension have been reported in many studies [21-23]. The present study found that hypertension prevalence declined after 80 years, the difference might partly be due to the shorter survival of people with AH and a low incident in the older age group. As demonstrated in this study, isolated systolic hypertension is the most common form of hypertension found in elderly [24]. The predominance of systolic hypertension may reflect the consequences of a tendency for clinicians to treat it less aggressively than diastolic hypertension.

### Awareness, treatment and control of hypertension

Only 19% of the respondents were newly diagnosed with hypertension. Although these proportions are quite similar to those reported from other developing countries [25]. Population studies have shown different rate of hypertension awareness by hypertensive individuals. In European countries, these levels range from 52.7% in Germany to 70% in Sweden. In Canada and USA the awareness rate is 83% and 87.3% respectively [26,27]. In these studies, the degree of awareness of hypertension was high (81%) than those reported by the India study [28]. Wyatt et al. reported that awareness significantly increased with age, female sex, presence of major co-morbidity and receiving preventive care [29], a combination of these factors contributes to the high level of awareness in our study.

Among treated hypertensive individuals in this study, about 30% had blood pressure controlled. These data are also similar to those reported by Liau et al. (34.3%) [23], but lower than those reported by Cipullo et al. (52.4%). In Europe the blood pressure controls rate range from 22.9% in Spain to 37.7% in England [26]. The favourable results in our study may be due to adequate public information about hypertension and availability of free government-supplied anti hypertensive drugs with relatively high access to health care and insurance coverage.

### Correlates of hypertension

Regarding the educational level, there are an inverse but not significantly association between schooling and AH, the small number of educated population may influence this result. Although, this finding was consistent with previous reports [30,31]. Cipullo et al. reported that the risk of hypertension was 2.8-times higher for those with lower schooling [26]. There is an intricate relationship between physical health and psychological status. Depression is known to have diverse effects on body functions. A French study of the elderly in the community revealed that hypertension was associated with anxiety but not depression [32]. Our study showed the lack of association between depression symptoms and incident hypertension.

The benefits of physical activity in the prevention and treatment of high blood pressure have been very well described [33]. In the present study, physical activity was not at a reduced risk for hypertension, but self-reported bias and interviewer bias can result in no differential misclassification.

The higher, but not significant, prevalence of hypertension in non smokers should be interpreted with caution since it is possible that part of the existing difference may be due to the high percentage of current smokers for male compared to female. No association between AH and marital status, these results are in accordance to what has been reported in other study [28]. In Martinique and China populations, the urban population exhibit a risk profile of hypertension [34,35], but not urban/rural differences were observed in our study, it is important to note that in our study in Monastir City, only 14% are from rural sites, the small number may influence statistics. The higher prevalence rates in hypertension among the illiterate and in individuals living in urban area are often being attributed to differences in life style, modernization, a shift from an agricultural to non agricultural economy, physical activity and occupation. Socioeconomic difference play, also, an important role in health conditions influencing different factors such as access to the health system, degree of information and understanding of medical conditions [36,37].

Similar to several population-based studies [26,38,39], it was observed that there is an important association between BMI and AH, as well as waist circumference. Overweight and obesity are actual risk factors for hypertension. The positive correlation between waist circumference and AH identifies a simple, low cost and easy-to-apply measure as an important marker for AH.

The strong association between diabetes mellitus and hypertension has been reported by numerous studies [28,40]. It is widely appreciated that hypertension increase the risk of activities of daily living and instrumental activities of daily living decline and have an important impact on the dependency categories [41], our data support this observation.

### Limitations of the study

Some limitations of this study should be mentioned: first the use of a single visit to ascertain hypertension status can result in an over-estimation of its prevalence. Other potential sources of bias include the self-reported hypertensive treatment by the participants. Treatment status was assessed by the question of whether or not the person was currently using antihypertensive drugs (within the last two weeks). No information on type and dosage of antihypertensive medication being used or medication compliance was collected from participants, nor were they asked about AH control measures other than pharmacotherapy. Therefore, "treatment" was restricted to the use of medication, without consideration of other, non pharmaceutical strategies such as dietary sodium restriction.

In this study, we did not assess the lipid profile and blood glucose measurement, a fact that did not allow us to evaluate the association between the metabolic changes and high BP.

We used BMI as a simple method to categorize people as "healthy weight," "overweight," or "obese". BMI formula may overestimates fatness in elderly because of loss of height resulting from vertebral compression (a problem commonly associated with aging). Knee-heel length and weight/knee height as alternative measurements in the elderly were not measured.

On the other hand, we described the prevalence of hypertension among the elderly in Monastir city, in order to provide estimates of the problem, this adding information about a group that is under researched. The use of a door-to door survey, the large sample size, adequate representation of women and the very old and a health professional for blood pressure measurement are strengths of our study.

## Conclusions

Our findings provide important information on the prevalence, awareness and control of hypertension. More than half of our study population was classified as hypertensive. The magnitude of arterial hypertension in elderly had important implications for both health care and health promotion and poses a major economic challenge. The public health community must be alerted to the emergence of arterial hypertension as an important health problem. Despite high prevalence in Monastir city, our study suggest that educational efforts, individual level strategies combined with population level efforts directed at increasing the levels of awareness and treatment and reducing the average blood pressure of the population. After the estimation of the prevalence of hypertension, the formulation of effective treatment strategies is very important. Consequently, it is important that health planners, clinicians and public health practitioners formulate country-specific guidelines according to health care system in developing countries and her economic realities.

## Competing interests

The authors declare that they have no competing interests.

## Authors' contributions

SoH initiated, directed and designed the research, contributed to data collection, interpretation of results and drafted the manuscript. SM participated in the recruitment of study subjects and collection epidemiological data and contributed to the preparation of the final manuscript. SH contributed to study design, to assembly of data and interpretation of results performed the statistical analysis. NK participated in the recruitment of study subjects and collection epidemiological data. MAF contributed to assembly of data and commented on the manuscript. SK contributed to study design interpretation of results and commented on the manuscript. MH and FB supervised the study including interpretation of results and preparation of the manuscript. All authors read and approved the final manuscript.

## Pre-publication history

The pre-publication history for this paper can be accessed here:

http://www.biomedcentral.com/1471-2261/11/65/prepub
